# Characteristics and FEA verification of the attraction between like magnetic poles

**DOI:** 10.1038/s41598-023-30611-1

**Published:** 2023-03-02

**Authors:** Christina H. Chen, Min Zou, Sijie Ran, Hui Meng, George Mizzell, Abby Shen, Michelle Qian

**Affiliations:** 1 Quadrant Solutions Inc., San Jose, CA 95131 USA; 2 Lab Magnetics Inc., San Jose, CA 95131 USA; 3 Foresee Group, Hangzhou, 311500 Zhejiang China; 4 SuperMagnetMan, Pelham, AL 35124 USA; 5 Quadrant International Inc., San Diego, CA 92121 USA

**Keywords:** Engineering, Materials science, Physics

## Abstract

The attraction between unequally sized like magnetic poles is characterized herein. Finite element analysis (FEA) simulation has verified that attraction can occur between like poles. Between two unequally sized like poles with various dimensions and alignments, a turning point (*TP*) appears on the curves of force vs. distance between them, which is caused by the localized demagnetization (*LD*). The *LD* plays a role far before the distance between the poles reduces to the *TP*. The *LD* area may have a changed polarity, making the attraction possible and not in violation of basic laws of magnetism. Here, the *LD* levels have been determined using FEA simulation, and the factors affecting the *LD* have been explored, including the geometry, the linearity of the BH curve, and the alignment of the magnet pairs. Novel devices can be designed with attraction between the centers of such like poles and repulsion when off-center.

## Introduction

The basic law of magnetism is described by Dr. Bozorth in his 1951 book^1^, where he states, “Poles exert forces on each other: north and south poles attract each other and like poles repel with a force that varies inversely as the square of the distance between them.” Recently, several reports have described an unusual magnetic interaction, in which two unequally sized like poles attract each other^2–5^. In 2021, we reported our research results in an article titled “Revealing the mystery of the cases where Nd-Fe-B magnetic like poles attract each other”^6^, where the attraction between like poles in the central area was attributed to the localized demagnetization (*LD*), based on the experimental data of the force vs. distance between poles and revelation of the magnets’ surface field. Usually, the repelling force of equally sized like poles is inversely proportional to the squared distance between them and thus monotonically increases as the distance decreases. However, unequally sized magnets have different permeance coefficient (*P*_*c*_) values; therefore, a magnet with a higher |*P*_*c*_| can locally demagnetize the other with a lower |*P*_*c*_|. The *LD* area may have a changed polarity, which forms an unlike pole region relative to the other magnet, resulting in an attracting force superimposed on the preexisting repelling force between them. When the distance becomes sufficiently small, the *LD* effect becomes strong enough for certain magnets in certain geometries, and then the attracting force overcomes the repelling force to form a net attracting force. Therefore, the basic law of magnetism is not violated. In the curves of force vs. distance between unequally sized like poles, when the distance decreases, the repelling force increases first and then decreases after passing a turning point (*TP*)^7^. The linearity of the demagnetization BH curve also plays an important role. The mechanism behind the attraction of the like poles has been described in Ref.^6^. However, to make practical use of this unusual phenomenon, it is necessary to obtain detailed characteristics of the attracting conditions for like magnetic poles with other alignment arrangements and to provide theoretical verification using finite element analysis (FEA). This article reports FEA simulation results using the newest version of a commercial software with advanced capability of treating permanent magnets using the whole non-linear BH curves. The FEA results agree well with the experimental data, thus successfully verified the observed attraction between like magnetic poles theoretically. Factors that determine the *LD* effect are further sorted out in this reported study. Magnetic field distributions in the space occupied by the magnetic pairs were calculated by FEA. The *LD* levels at different configurations of magnetic pairs (i.e., distance between the magnets, magnets’ dimensions, and their alignment arrangements) were determined quantitatively based on the magnetic field distributions. These in-depth understandings beyond the prior work^6^ pave the way for developing novel magnetic devices.

## Results

### Factors that determine the *LD* effect of center and edge aligned unequally sized magnet pairs

**Figure 1:**
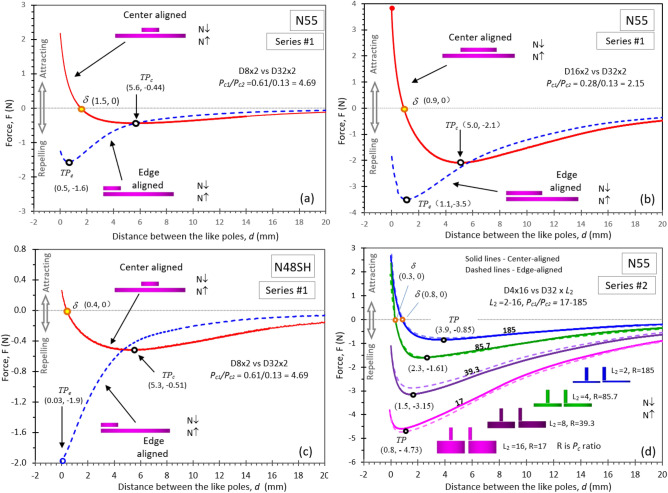
The curves of force vs. distance between like poles show attraction at a sufficiently small distance for NdFeB pairs. In Series #1 (thin magnet pairs), the pairs of N55 magnets with *P*_*c*_ ratios of 10.8 (not shown here), 4.69 (**a**), and 2.15 (**b**), all show the transition from a repelling force to an attracting force for the center-aligned pairs, but such a transition is absent for the edge-aligned pairs. The N48SH pairs with *P*_*c*_ ratios of 4.69 (**c**) and 10.8 (not shown here) also exhibit the same features as the N55 pairs. This indicates that the *LD* effect is stronger in the center-aligned than in the edge-aligned for the thin magnet pairs. In Series #2 (thick magnet pairs), the pairs of N55 with *P*_*c*_ ratios of 17 to 185 show negligible force differences between the center-aligned and edge-aligned (**d**). Two pairs with larger *P*_*c*_ ratios of 85.7 and 185 show the transition from repelling to attracting, and the other two pairs with smaller *P*_*c*_ ratios of 17 and 39.3 do not have such a transition.

Fourteen curves of magnetic force vs. distance between poles for NdFeB N55 and N48SH magnet pairs of the center- and edge-aligned like poles are shown in Fig. 1. All the curves exhibit a *TP*, which is caused by the *LD* effect of the like poles of unequal size^6^. Figures 1(a) to (c) show a clear difference in the forces between the center- and edge-aligned pairs in Series #1 (see “Methods” section). The center-aligned pairs for both N55 and N48SH have a value of *d*_*TP*_ from 5.0 to 5.6 mm (where *d*_*TP*_ is the distance at the *TP*) and a *δ* point at *d*_*δ*_ from 0.4 to 1.5 mm (*δ* point is where the net force becomes zero). When the distance is smaller than *d*_*δ*_, the net force becomes an attracting force due to the *LD* effect.

**Figure 2:**
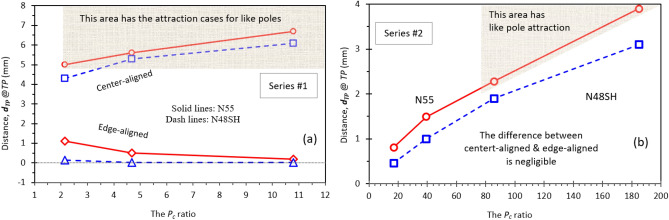
The relationship of the distance *d*_*TP*_ at *TP* and the *P*_*c*_ ratio. (**a**) Series #1 (thin magnet pairs) and (**b**) Series #2 (thick magnet pairs). A larger *d*_*TP*_ indicates a stronger *LD* effect and a greater possibility of attraction between the like poles. In Series #1(thin magnet pairs) both N55 and N48SH show a large difference in *d*_*TP*_ between the center- and edge-aligned. In Series #2 (thick pairs) have negligible differences between the two alignments.

Differing from the distinct transition from repulsion to attraction observed between the center-aligned like poles with a decreased distance, the forces between the Series #1 edge-aligned poles remain repulsive in all the measured distance ranges, as shown in Figs. 1(a) to (c). Since the attractive force between the like poles is caused by the *LD* effect^6^, the absence of the attractive force in the edge-aligned suggests a weaker *LD* effect in the edge-aligned than in the center-aligned in Series #1. The weaker *LD* effect is also manifested in the smaller *d*_*TP*_ of the edge-aligned, with *d*_*TP*_ = 0.03 to 1.1 mm, which is much smaller than the *d*_*TP*_ in the center-aligned, as shown in Figs. 1 and 2. Therefore, a larger d_*TP*_ implies a stronger *LD* effect and an increased possibility of attraction between the like poles.

Contrary to Series #1, the force difference between the center- and edge-aligned poles is negligible for all the Series #2, N55 tested pairs, as illustrated in Fig. 1(d). The two center-aligned Series #2 pairs with *P*_*c*_ ratios of 85.7 and 185 exhibit the force transition from repulsion to attraction at *d*_*δ*_ values of 0.3 and 0.8 mm, respectively. The two edge-aligned pairs with the same *P*_*c*_ ratios also exhibit the same force transition at the same *d*_*δ*_. Since the *P*_*c*_ ratios of 85.7 and 185 are the highest among all the pairs measured in this study, a higher *P*_*c*_ ratio evidently leads to a stronger *LD* effect.

Figure 2 shows the relationship of the distance *d*_*TP*_ at *TP* and the *P*_*c*_ ratio of Series #1 (thin magnet pairs) and Series #2 (thick magnet pairs). A larger *d*_*TP*_ indicates a stronger *LD* effect and a greater possibility of attraction between the like poles. The shaded areas with larger *d*_*TP*_ in Fig. 2(a) and (b) show the attraction cases for the like poles. In Series #1, the pairs of both N55 and N48SH show a large difference in *d*_*TP*_ between the center- and edge-aligned pairs. The force difference between the center- and edge-aligned N55 pairs, as well as N48SH pairs is negligible in Series #2. Additionally, N48SH has a weaker *LD* effect in both series, which is evident by (i) the absence of the transition from repulsion to attraction in the whole distance range for most of the pairs and (ii) smaller *d*_*TP*_ values at the *TP*s. As shown in the “Methods” section Fig. 7, N48SH has a linear BH curve in the 2nd quadrant and part of the 3rd quadrant, which provides N48SH with stronger resistance to demagnetization and, in turn, a weaker *LD* effect. It is evident that the attraction likely occurs for the like poles when the *P*_*c*_ ratios are higher. For Series #1, when *P*_*c*_ ratios > 2, the attraction occurs for all the center-aligned like magnetic poles.

### FEA simulation verification of the LD effect and the attraction between the like poles

Simcenter MagNet 2022.1 was used for FEA to simulate the interacting forces of the like magnetic poles. This newest version of software enables the actual BH data in the 2nd and 3rd quadrants to be adapted, providing higher simulation accuracy for short magnets (i.e. small |*P*_*c*_|) with nonlinear BH curves, such as NdFeB N55. The interaction of like poles across a small distance, or for some poles in dynamic applications, involves the demagnetizing field that reaches the 3rd quadrant^8^.

**Figure 3:**
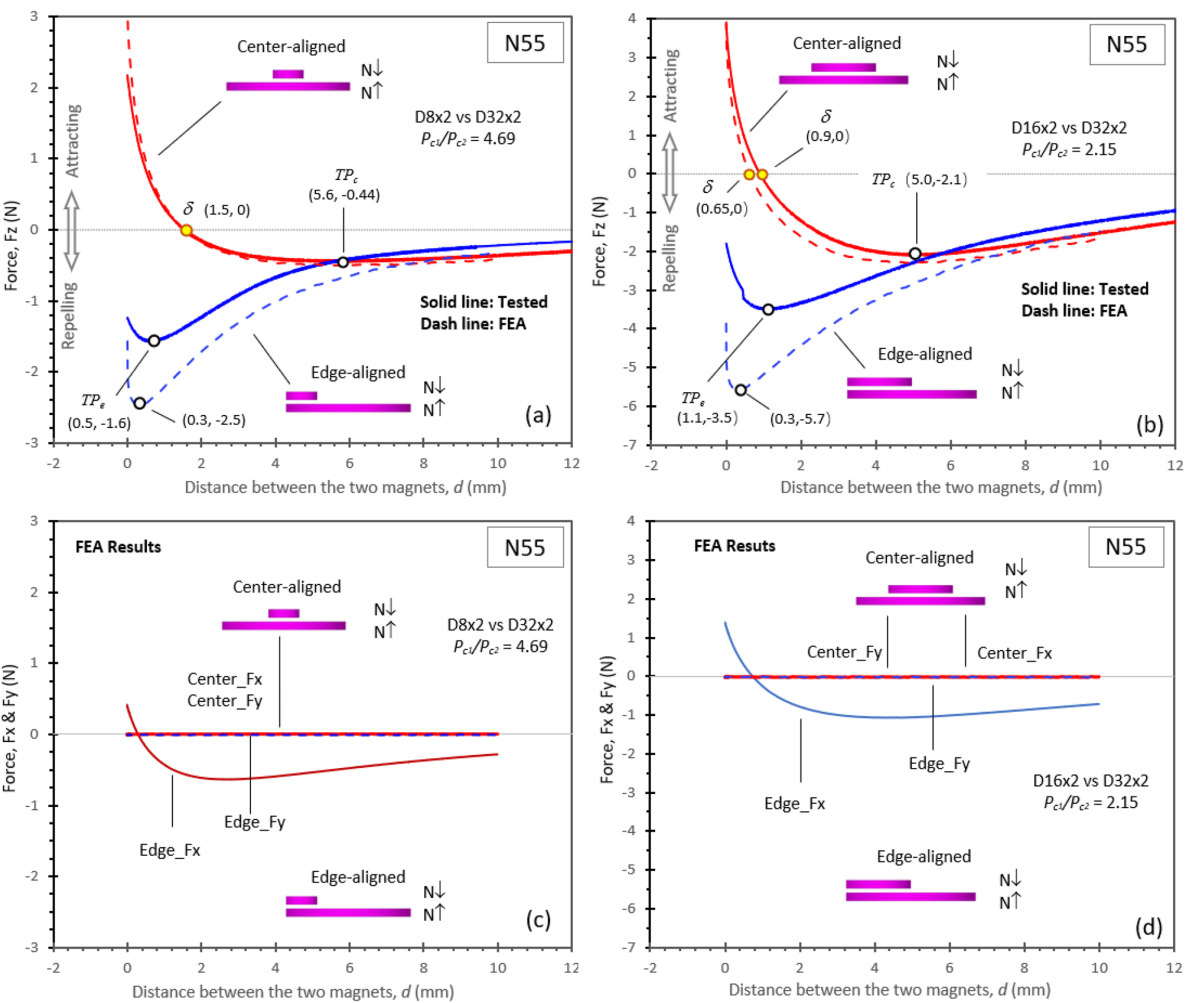
The tested force and FEA simulated force *F*_z_ vs. distance between two sets of N55 like poles. For the center-aligned like poles, the tested and simulated results are almost identical, with the same turning points *TP* and the zero force points δ. All these four curves of force vs. distance shown in Fig. 3(**a**) and (**b**) display an undisputable transition from a negative force, or a repulsion, to a positive force, or an attraction. This is a remarkable verification of the fact that the attraction can occur to these like magnetic poles. For the edge-aligned like poles, although the tested and simulated forces *F*_z_ shown in Fig. 3(**a**) and (**b**) do not completely coincide, the shapes of the curves are similar. Both the tested and simulated curves show the turning point *TP* at similar distances. It is believed that the difference between the tested *F*_z_ and the FEA simulated *F*_z_ for the edge-aligned pairs is due to the complication or the influence from the force in the x-axis direction, *F*_x_. As shown in Fig. 3(**c**) and (**d**), the *F*_x_ is non-neglectable, and it produces a torque to affect the *F*_z_. The fact of the turning point *TP* is unmistakable, and the simulated curves show a larger repelling force. The FEA successfully verified the attractive condition for unequally sized like poles.

Using the same magnet pairs which were tested for the interactive force, with the same BH data in the 2nd and 3rd quadrants of the magnets, FEA 3D models were set with meshes of 0.5 mm in the targeted areas. The magnetic directions for the two like magnet poles were set opposite to each other. The simulations resulted in the interactive forces between the poles. More details of FEA setup can be read in the “Methods” section. The tested curves and the FEA simulated curves of two pairs of N55 like poles with different *P*_*c*_ ratios are shown in Fig. 3 for comparison and verification. For the two sets of N55 like poles, D8 × 2 vs. D32 × 2 and D16 × 2 vs. D32 × 2, the center-aligned like poles’ tested results and the FEA simulated plots are almost identical. For the pair of D8 × 2 vs. D32 × 2 as shown in Fig. 3(a), both the tested and simulated results show the same *TP*_*c*_ at *d* = 5.6 mm and *F* = − 0.44 N, and the same force zero-point *δ* at *d* = 1.5 mm. For the pair of D16 × 2 vs. D32 × 2 as shown in Fig. 3(b), both the tested and simulated results show the same *TP*_*c*_ at *d* = 5.0 mm and *F* = − 2.1 N, and slightly different force zero-points: the *δ* is at *d* = 0.9 mm on the tested curve, and the *δ* is at *d* = 0.65 mm on the FEA simulated curve. All these four curves of force vs. distance show an undisputable transition from a negative force, or a repulsion, to a positive force, or an attraction. This is a remarkable verification of the fact that the attraction can occur to these like magnetic poles.

For the edge-aligned like poles, although the tested and the FEA simulated force *F*_z_ shown in Figs. 3(a) and (b) do not completely coincide, the shapes of the curves are similar. It is believed that the difference between the tested *F*_z_ and the FEA simulated *F*_z_ for the edge-aligned pairs is related to the influence from the force *F*_*x*_, which is the force along the x-axis direction. As shown in Figs. 3(c) and (d), the *F*_x_ is non-neglectable, and it would produce a torque to affect the *F*_z_. This can explain why there is a force difference between the tested force and simulated force, as the simulated force *F*_z_ cannot include influences from other directions. Both the tested and simulated results show the turning point *TP* at similar distances. For the pair of D8 x 2 vs. D32 × 2, the tested *TP*_*e*_ is (0.5, − 1.6), and the simulated *TP*_e_ is (0.3, − 2.5), as shown in Fig. 3(a). For the pair of D16 × 2 vs. D32 × 2, the tested *TP*_*e*_ is (1.1, − 3.5), and the simulated *TP*_e_ is (0.3, − 5.7), as shown in Fig. 3(b). The fact of the turning point *TP* is unmistakable, even the simulated curves show a larger repelling force. Therefore, the FEA simulation successfully verified the attractive condition for unequally sized like poles. This verification provides confidence for the future design of devices based on this simulation model.

### Determining the LD level (LDL) using the magnetic field distributions simulated from FEA

**Figure 4:**
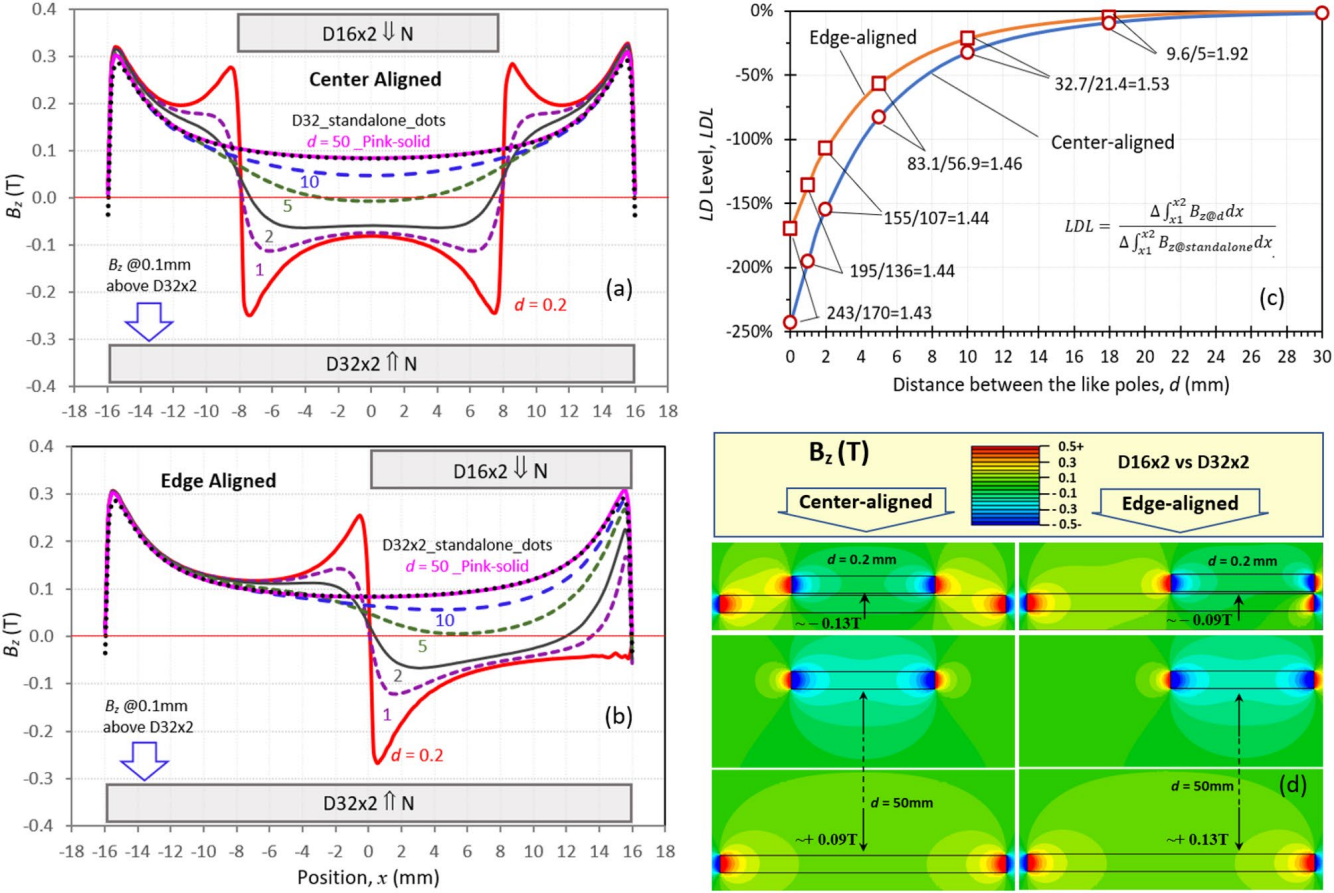
The B_z_ distributions of the center-aligned D16 × 2 and D32 × 2 pair with various distances show that the smaller the distance is, the stronger the *LD* and the *EE* (edge effect) are. The *LDL* is defined as the ratio of $\Delta {\int }_{{x}_{1}}^{{x}_{2}}{B}_{z}dx$ at the distance *d* over the ${\int }_{{x}_{1}}^{{x}_{2}}{B}_{z}dx$ of a standalone D32 × 2, calculated using Eq. (1), with the details for this case shown here: $LDL=\frac{\Delta {\int }_{{x}_{1}}^{{x}_{2}}{B}_{z@d}dx}{{\int }_{{x}_{1}}^{{x}_{2}}{B}_{z@\mathrm{standalone}}dx}=\frac{{\int }_{{x}_{1}}^{{x}_{2}}{B}_{z@\mathrm{distance}\_d}dx-{\int }_{{x}_{1}}^{{x}_{2}}{B}_{z\_D32x2\_\mathrm{standalone}}dx}{{\int }_{{x}_{1}}^{{x}_{2}}{B}_{z\_D32x2\_\mathrm{standalone}}dx}$, where *x*_1_ and *x*_2_ are the positions where the *LD* starts and ends. Plots for the center aligned pairs have ${x}_{1}=-8\mathrm{mm}$ and ${x}_{2}=8\mathrm{mm}$ (**a**), and for the edge aligned pairs have ${x}_{1}=0\mathrm{mm}$ and ${x}_{2}=16\mathrm{mm}$ (**b**). The *|LDL|* of the center-aligned pairs is as large as 243% at d = 0.2 mm, and the center-aligned pairs have much larger |*LDL*| than the edge-aligned pairs. The ratios of *|LDL|*_cente*r*_/*|LDL|*_edge_ range from 1.43 to 1.92 for *d* = 0.2 to 18 mm (**c**). The 2D *B*_*z*_ maps at the cross-section through a diameter for both center- and edge-aligned with *d* = 0.2 and 50 mm are displayed in (**d**), showing the *LD* and the *EE*s.

**Figure 5:**
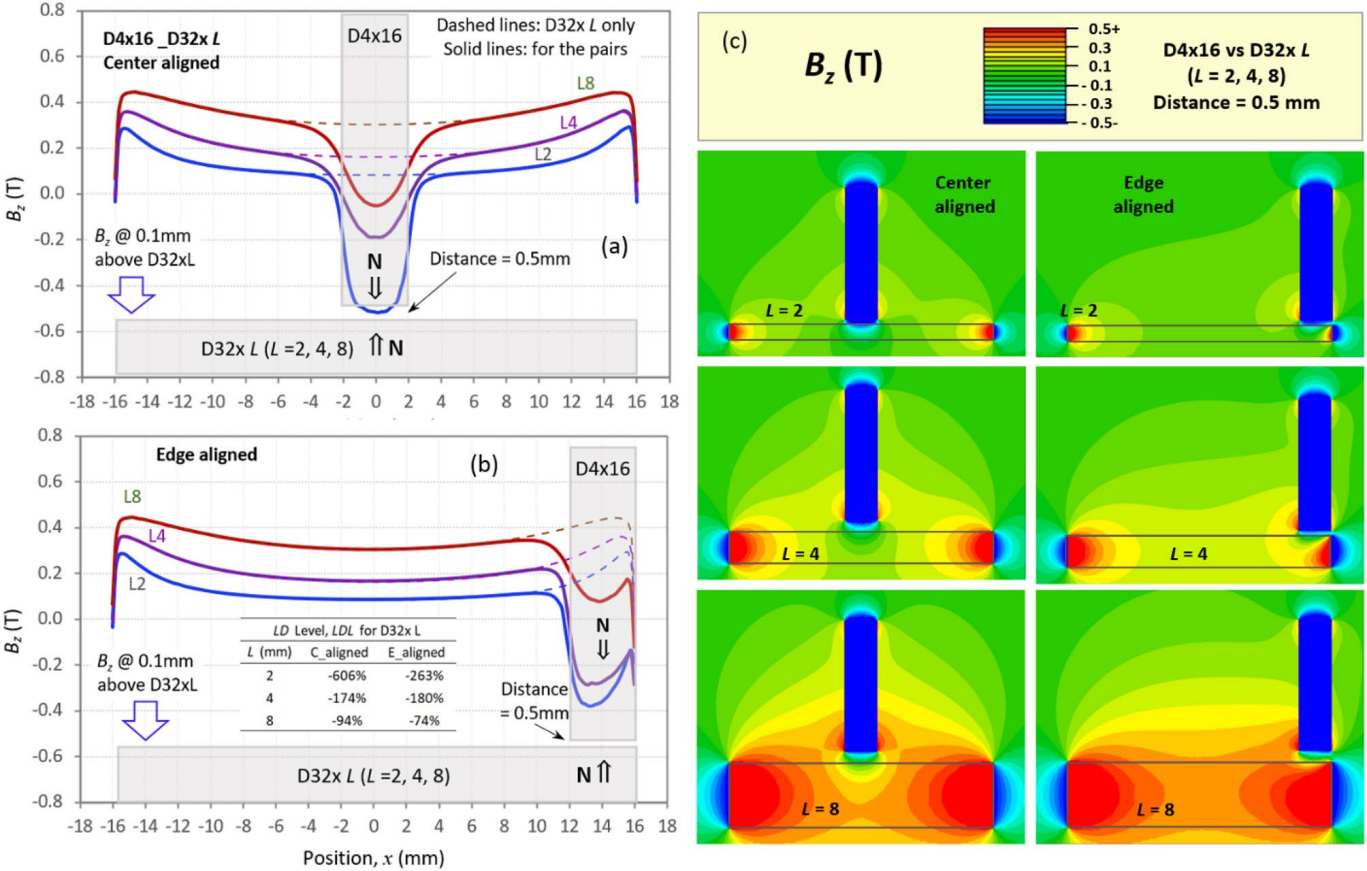
The *B*_*z*_ distributions show the *LDL* and the *EE* of three N55 pairs of D4 × 16 vs. D32xL like poles in Series #2 for the center-aligned (**a**) and edge-aligned (**b**). The *LDL* values of D32xL are listed in the inset of (**b**), which were calculated by using Eq. (1). The details for this individual case are shown here: $LDL=\frac{\Delta {\int }_{{x}_{1}}^{{x}_{2}}{B}_{z\_\mathrm{D}32\mathrm{x}L+\mathrm{D}4\mathrm{x}16}dx}{{\int }_{{x}_{1}}^{{x}_{2}}{B}_{z\_\mathrm{D}32\mathrm{x}L }dx}=\frac{{\int }_{{x}_{1}}^{{x}_{2}}{B}_{z\_\mathrm{D}32\mathrm{x}L+\mathrm{D}4\mathrm{x}16}dx-{\int }_{{x}_{1}}^{{x}_{2}}{B}_{z\_\mathrm{D}32\mathrm{x}L }dx}{{\int }_{{x}_{1}}^{{x}_{2}}{B}_{z\_\mathrm{D}32\mathrm{x}L }dx}$, where ${B}_{z\_\mathrm{D}32\mathrm{x}L+\mathrm{D}4\mathrm{x}16}$ is the *B*_*z*_ of D32x*L* paired with D4 × 16, and ${B}_{z\_\mathrm{D}32\mathrm{xL}}$ is the *B*_*z*_ of the standalone D32x*L*. The *x*_1_ and *x*_2_ are the positions enclosing the *LD* effect regions beneath D4 × 16. In the table, the column headers “C_aligned” and “E_aligned” stand for center-aligned and edge-aligned, respectively. Plots are given for the center-aligned pairs ${x}_{1}=-2\mathrm{mm}$ and ${x}_{2}=2\mathrm{mm}$ (**a**) and for the edge aligned pairs ${x}_{1}=12\mathrm{mm}$ and ${x}_{2}=16\mathrm{mm}$ (**b**). The 2D *B*_*z*_ maps at the cross-sections through diameters of the center- and edge-aligned three pairs are shown in (**c**).

**Figure 6:**
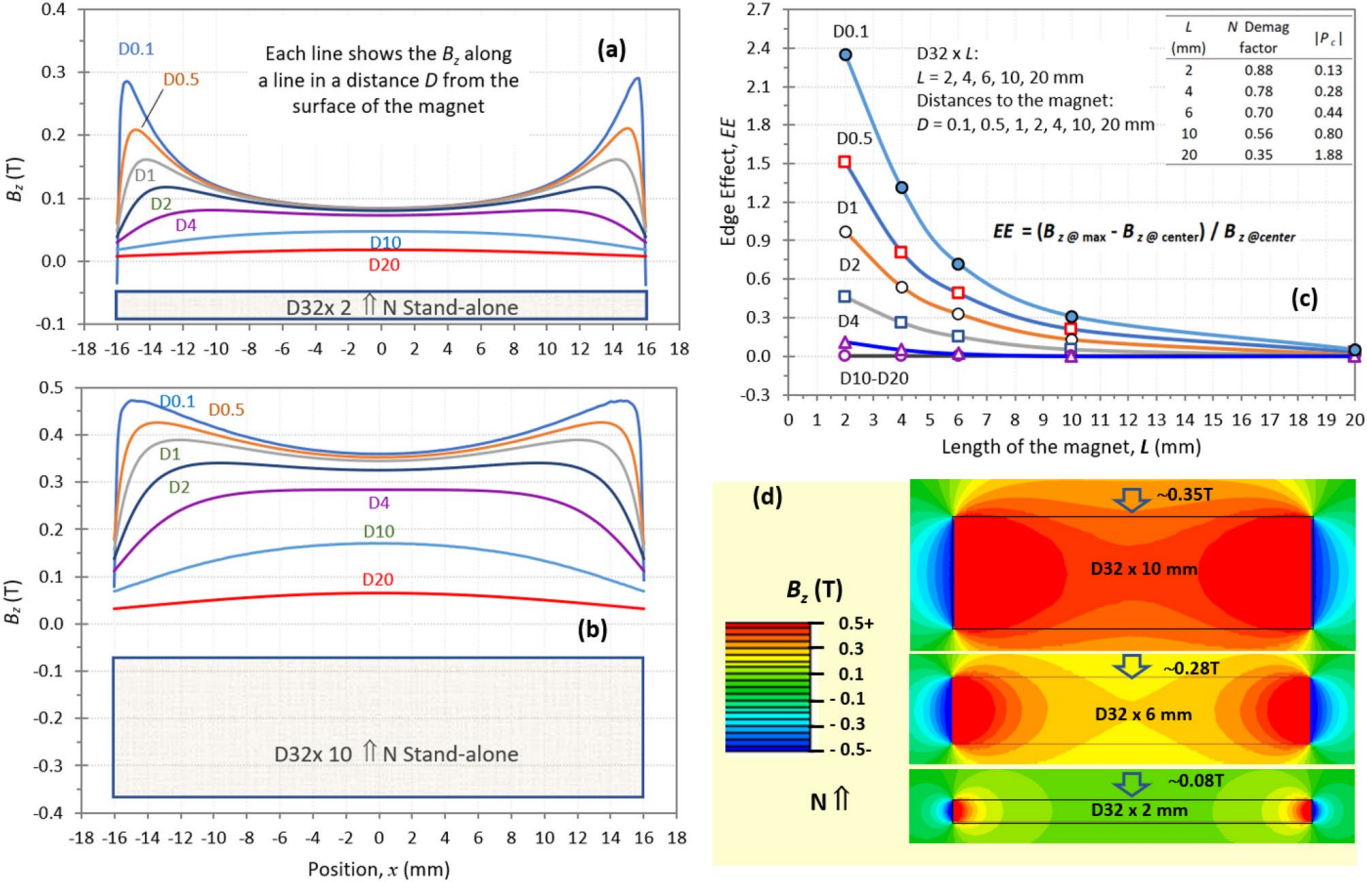
The FEA simulation shows the self-demagnetization and the *EE* vs. magnet length for a series of stand-alone N55 magnets with the same diameter and five lengths. The *B*_*z*_ vs. *x* of D32 × 2 is shown in (**a**), and the *B*_*z*_ vs. *x* of D32 × 10 is shown in (**b**). The *EE* values vs. magnet length and distance are shown in (**c**), and *EE* is defined as the difference between the maximum *B*_*z*_ and the center *B*_*z*_ over the center *B*_*z*_ on the curve of *B*_*z*_ vs. *x*: $EE\, = \,\left( {B_{{\text{z@max}}} \, - \,B_{{\text{z@center}}} } \right)\,/\,B_{{\text{z@center}}}$. When the maximum *B*_*z*_ is at the center, the *EE* becomes zero. The *EE* is 2.4 for the magnet of *L* = 2 mm, meaning the edge’s maximum *B*_*z*_ is 240% higher than that of the center *B*_*z*_. The 2D *B*_*z*_ maps at the cross-section through a diameter of the three magnets are shown in (**d**), in which the center *B*_*z*_ of 0.35 T at *L* = 10 mm is over four times the center *B*_*z*_ of 0.08 T at *L* = 2 mm.

The *LD* affects the interaction force far before the distance *d* decreases to *d*_*TP*_. At *d*_*TP*_, the *LD* is strong enough to generate an attracting force that can offset the increase in the repelling force as *d* decreases, resulting in the curve reaching its extreme, and then the repelling force decreases at *d* < *d*_*TP*_. For some magnet pairs, the net force becomes zero at *d* = *d*_*δ*_ and then becomes an attracting force at *d* < *d*_*δ*_. To determine how and where the *LD* affects the interacting magnetic force, FEA was used to simulate the distributions of the magnetic induction *B* in the distances, since the force *F* is proportional to *B*^*2*^ (*F* = *aA⋅B*^*2*^, where “*a*” is a constant and “*A*” is the area of the magnet)^9–12^. Figures 4, 5 and 6 show the distributions of the FEA-simulated *B*_*z*_ for representative magnets, where *B*_*z*_ is the *B* vector component along the magnets’ axial direction.

Figure 4 shows the simulated *B*_*z*_ vs. the position *x* for the like poles of D16 × 2 vs. D32 × 2 and the layout of the magnets (this is the same pair shown in Fig. 1(b). The *B*_*z*_ is calculated along a line 0.1 mm above a diameter of D32 × 2. Figure 4(a) displays the *B*_*z*_ in the center-aligned pair with six distances between the like poles (*d* = 0.2, 1, 2, 5, 10, and 50 mm). For a standalone D32 × 2, the *B*_*z*_ values are positive across the whole range of position *x* from − 16 to + 16 mm, showing up as black dots, almost overlapping on the curve of *B*_*z*_ at *d* = 50 mm. Pairing with like poles, the *B*_*z*_ vectors of D32 × 2 and D16 × 2 are opposite, thus repelling each other. As D16 × 2 has a higher |*P*_*c*_| (0.28) than D32 × 2’s |*P*_*c*_| (0.13), the stronger fluxes of D16 × 2 push the *B*_*z*_ near D32 × 2 from positive to negative when the distance *d* is ≤ 5 mm. At *d* = 0.2 mm, the edge fluxes of D16 × 2 give a powerful push, making the curve of *B*_*z*_ vs. *x* near D32 × 2 look like a pair of upside-down cat ears. Figure 4(b) shows the *B*_*z*_ of edge-aligned like poles, and the *LD* effect is weaker than that of the center-aligned poles. At *d* = 0.2 mm, the *B*_*z*_ curve is in the shape of only one cat ear.

The *LD* level, *LDL,* of D32 × 2 for both center- and edge-aligned cases is shown in Fig. 4(c). The *LDL* is defined as the ratio of $\Delta {\int }_{{x}_{1}}^{{x}_{2}}{B}_{z}dx$ at the distance *d* over ${\int }_{{x}_{1}}^{{x}_{2}}{B}_{z}dx$ of the standalone D32 × 2, and the standalone magnet is used as the base. The following equation is established to calculate the *LDL*, and the details of this individual case are given in the caption of Fig. 4.

1 $$LDL=\frac{\Delta {\int }_{{x}_{1}}^{{x}_{2}}{B}_{z@d}dx}{{\int }_{{x}_{1}}^{{x}_{2}}{B}_{z@base}dx}=\frac{{\int }_{{x}_{1}}^{{x}_{2}}{B}_{z@d}dx-{\int }_{{x}_{1}}^{{x}_{2}}{B}_{z@base}dx}{{\int }_{{x}_{1}}^{{x}_{2}}{B}_{z@base}dx}$$

The *LDL* plays a role far before the distance reduces to the *TP*. As shown in Fig. 4(c), when the distance *d* increases, |*LDL*| decreases, but even at *d* > 10 mm, *LDL* is nonnegligible. For example, for the center-aligned pairs, |*LDL*| is 32.7% at *d* = 10 mm and 9.6% *at d* = 18 mm. When near the *TP*, |*LDL*| is 83.1% at *d* = 5 mm. At *d* = 0.2 mm, |*LDL*| is as large as 243%.

The |*LDL*| of the center-aligned poles is higher than that of the edge-aligned poles, and the ratios of |*LDL*|_center_/|*LDL*|_edge_ range from 1.43 to 1.92 in the distances of 0.2 to 18 mm. This is in good agreement with the difference in the force vs. distance behaviors between the center- and edge-aligned pairs, as shown in Figs. 1, 2 and 3, where the force on the center-aligned Series #1 N55 pairs and some N48SH pairs transforms from repulsive to attractive with sufficiently small distances, while the force on the edge-aligned pairs remains repulsive in the same distance range.

The 2D *B*_*z*_ maps at the cross-section through a diameter for both center- and edge-aligned like poles with distances *d* = 0.2 and 50 mm are displayed in Fig. 4(d), showing the *LD* effect and edge effect (*EE*, i.e. higher *B*_*z*_ fluxes near the edges). The curves of *B*_*z*_ vs. *x* at the distances below D16 × 2 are also affected by D32 × 2, but the *B*_*z*_ near D16 × 2 remains negative without changing the polarity of D16 × 2 since it has a higher *P*_*c*_.

The *B*_*z*_ distributions of three pairs of Series #2 N55 like poles are shown in Fig. 5, which have the same dimensions as three of the four shown in Fig. 1(d); these are D4 × 16 vs. D32x*L* (*L* = 2, 4, and 8 mm), with *P*_*c*_ ratios of 185, 85.7, and 39.3 and a fixed distance of 0.5 mm. The *B*_*z*_ is calculated along a line 0.1 mm above a diameter of D32x*L*. Figure 5(a) and (b) show the *B*_*z*_ of center- and edge-aligned pairs, respectively. The *B*_*z*_ curves of the standalone D32x*L* are displayed in the figures as dashed lines. The *LDL* values are listed in the inset of Fig. 5(b), which were calculated by using Eq. (1). The calculation details for this individual case are shown in the caption of Fig. 5.

The |*LDL*| values of the center- and edge-aligned are close to each other for D32 × 4 and D32 × 8, which are in good agreement with the similarity of the force vs. distance behaviors between the two alignment conditions, as shown in Fig. 1(d). The |*LDL*| values for D32 × 2, on the contrary, are substantially different between the center- and edge-aligned (606 vs. 263%, respectively), showing a much stronger *LD* effect in the center-aligned than in the edge-aligned. The remarkable difference in |*LDL*|, however, does not lead to a distinct difference in their force vs. distance behaviors. This is thought to be because the *LD* effect on the force saturates when |*LDL*|> 260%.

The edge and *LD* effects are exhibited in the 2D *B*_*z*_ maps at the cross-sections through diameters of the three center- and edge-aligned pairs, as shown in Fig. 5(c). A shorter magnet length *L* results in a stronger *LD* effect, as it has a weaker resistance to demagnetization. D4 × 16 has a very high *P*_*c*_ (= 24), acting like a pushpin. Differing from the *B*_*z*_ distributions shown in Fig. 4 for D16 × 2 and D32 × 2 in Series #1, where D16 × 2 (*P*_*c*_ = 0.28) pushes the *B*_*z*_ down to take the shape of upside-down cat ears, D4 × 16 in Series #2 pushes the *B*_*z*_ down to a hole that has a shape of an upside-down Gaussian distribution.

To further explore the *LD* and *EE*s (edge effects), the *B*_*z*_ distributions of a set of standalone N55 magnets of D32x*L* (*L* = 2, 4, 6, 10, and 20 mm) are simulated, with the magnetization direction along the length. The curves of *B*_*z*_ vs. position *x* of D32 × 2 and D32 × 10 are displayed in Figs. 6(a) and (b), with seven curves in each figure representing the *B*_*z*_ along a line parallel to the diameter at seven distances *D* (0.1 to 20 mm) from the magnets. At a short distance of *D* ≤ 2 mm, the magnets have a large self-demagnetization with a lower *B*_*z*_ at the centers and a much higher *B*_*z*_ near the edges. The *EE* is defined as the difference between the maximum *B*_*z*_ and the center *B*_*z*_ over the center *B*_*z*_ on the curve of *B*_*z*_ vs. *x*:

2 $$EE\, = \,\left( {B_{{\text{z@max}}} \, - \,B_{{\text{z@center}}} } \right)\,/\,B_{{\text{z@center}}}$$

When the maximum *B*_*z*_ is at the center, the *EE* becomes zero. The *EE* relates to self-demagnetization and the *LD* strength when pairing with other magnets. A magnet with a higher *EE* has a lower |*P*_*c*_|, signifying a stronger *LD* effect when paired with a magnet with a higher |*P*_*c*_|. Figure 6(c) exhibits the *EE* vs. the magnet length at seven distances, with the |*P*_*c*_| and demagnetization factor *N* of these magnets shown in the inset. For all five magnets, when *D* is 10 mm or larger, *EE* = 0, showing the maximum *B*_*z*_ at the center. When the distance reduces from 10 to 0.1 mm, the *EE* increases from 0 to 2.4 for the magnet of *L* = 2 mm, meaning the edge’s maximum *B*_*z*_ is 240% higher than the center *B*_*z*_. A larger *EE* represents a stronger *LD* effect near the center. For a distance of *D* = 0.1 mm, the *EE* decreases rapidly with the length of the magnets. For the magnet with 20 mm length, the *EE* is closed to zero for all the distances*.* Even for the smallest distance of 0.1 mm, the *EE*_*L20@0.1*_ = 0.05. Figure 6(d) shows 2D *B*_*z*_ maps at the cross-section along a diameter of the D32 × 2, D32 × 6, and D32 × 10 magnets. For the same grade N55, the *B*_*z*_ on the center surface is 0.08 T on D32 × 2 and 0.35 T on D32 × 10. The conclusion from the data shown in Fig. 6 is that a magnet with a short length can have a stronger *EE* with higher *B*_*z*_ on edges, which implies a stronger *LD* effect when paired with other magnets having a higher |*P*_*c*_|.

### Summary

The attraction between unequally sized like magnetic poles has been characterized for those aligned at not only the centers but also the edges. FEA simulation has verified that the attraction can occur with like poles. For all unequally sized like poles aligned at the centers and edges, a *TP* appears on the curves of force vs. distance between poles, caused by *LD*. The *LD* area may have a changed polarity, and a strong *LD* effect can result in attraction in certain like poles without violating the basic law of magnetism. The characteristics of the attraction between like poles include the following:

- (i) The localized demagnetization *LD* plays a role far before the distance reduces to the turning point *TP*. A stronger *LD* effect leads to a larger *d*_*TP*_ and a higher possibility of attraction between like poles.
- (ii) The LD levels *LDL*s can be predicted using the magnetic flux distribution from FEA simulations.
- (iii) A magnet with a short length (a low |*P*_*c*_|) when paired with another magnet with a high |*P*_*c*_| has a large edge effect *EE*, implying a strong *LD* effect when paired with another thicker magnet (a higher |*P*_*c*_|).
- (iv) NdFeB N55 has a stronger *LD* effect than N48SH or other high coercivity grades because N55’s BH curve is nonlinear in the 2nd quadrant.
- (v) For magnet pairs with short lengths (such as those in Series #1), the *LD* effect of the center-aligned like poles is stronger than that of the edge-aligned poles. Novel applications can use this feature of attraction when on-center and repulsion when off-center. For magnet pairs with long lengths (such as those in Series #2), the difference in the *LD* effect between the center- and edge-aligned like poles is negligible. Novel applications can also be designed with attraction in a small distance and repulsion in an increased distance. Research on novel applications is underway.

## Conclusions

Finite element analysis (FEA) simulation has theoretically verified that attraction can occur between magnetic like poles. FEA has also simulated magnetic field distributions in various conditions. The *LD* levels have been quantitatively determined based on the magnetic field distributions. The factors affecting the *LD* have been sorted, including the geometry and the *P*_*c*_ ratio, the linearity of the BH curve, and the alignment methods of the magnet pairs. The attracting occurs for the like poles when the *P*_*c*_ ratios are higher than about 2 for the thin magnets (thickness ~ 2 mm). Novel devices can be designed with attraction between the centers of such like poles and repulsion when off-center.

## Methods

### Materials

**Figure 7:**
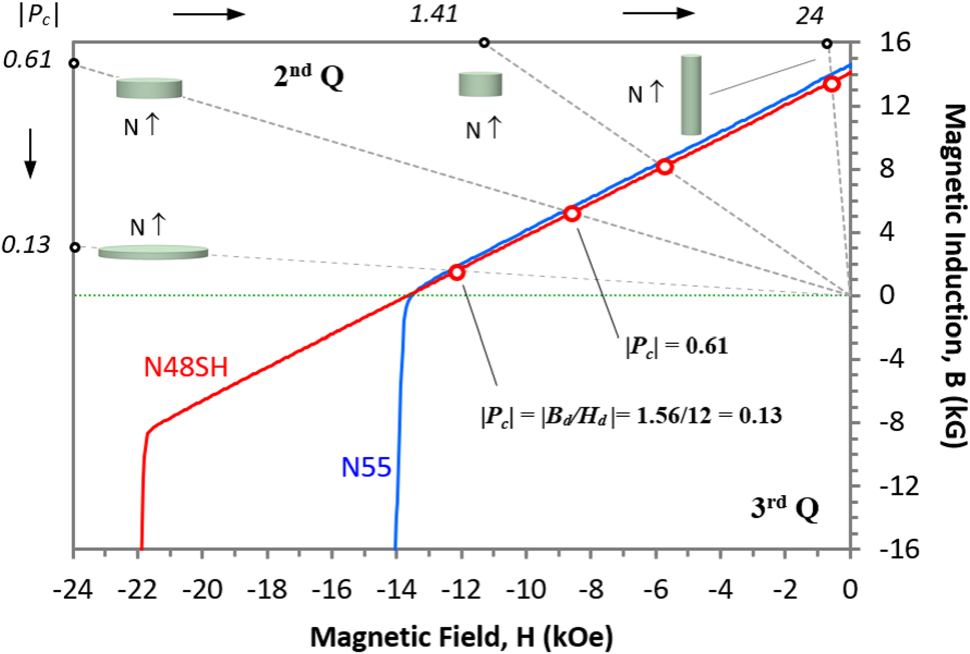
The demagnetization BH curves of N55 and N48SH, and the definition of permeance coefficient *P*_*c*_.

NdFeB N55 and N48SH magnet samples were tested in pairs with unequally sized like poles facing each other. The details, including the dimensions and the *P*_*c*_ values, were described previously^6^. The demagnetization BH curves of N55 and N48SH are shown Fig. 7, as well as the definition of permeance coefficients *P*_*c*_. The BH curve of N55 is nonlinear in the 2nd and 3rd quadrants, and that of N48SH is linear in the 2nd quadrant and part of the 3rd. These cylindrical samples are grouped in two series with various |*P*_*c*_| values from 0.13 to 24. In Series #1, all the magnets in three pairs have the same length of 2 mm, and the larger magnets with a lower |*P*_*c*_| have the same diameter of 32 mm (D32 × 2). The diameters of 4, 8, 16 mm of the small magnets result in the *P*_*c*_ ratios of 10.8, 4.69, and 2.15, respectively. In Series #2, all the higher |*P*_*c*_| small magnets have the same diameter of 4 mm with a length of 16 mm (D4 × 16), and all the lower |*P*_*c*_| large magnets have a diameter of 32 mm with lengths *L* = 2, 4, 8, and 16, resulting in the *P*_*c*_ ratios of 17, 39.3, 85.7, and 185, respectively. Both series are illustrated in the insets of Fig. 7.

### Force tests

The magnet pairs were tested for their interaction forces using an Instron Single Column Force Tester Model 5944. The test instrument has been calibrated regularly by an ISO 9001:2015 certified lab, with a force measurement uncertainty of 0.4% prior to these tests. Each pair of two magnets were first magnetized into full saturation. One magnet in the pair was then loaded in the upper sample mounting fixture, another magnet in the lower mounting fixture of the force tester, with the two magnets’ north poles facing each other. A high precision force sensor was attached to the upper sample mounting fixture. The gravity forces of the upper sample mounting fixture and the containing magnet were zeroed out when the two magnets were 100 mm vertically apart. The force measurements were started when the upper and lower magnets were 50 mm vertically apart. The upper magnet was vertically moved towards the lower magnet by the force tester at a constant speed of 5 mm/minute. The force sensor then measured the vertical forces between the two magnets as a function of distance between them from 50 to 0 mm. The measurement sequence and data collection were controlled by the force tester’s computer software Bluehill^®^ Universal.

The tests were carried out for both the center- and edge-aligned pairs, as shown in the insets of Fig. 1. The curves of measured force vs. distance for center-aligned pairs were reported previously^6^, and the measured curves for the edge-aligned pairs are reported for the first time here.

### Permeance coefficient *P*_*c*_ calculation

All the *P*_*c*_ values were calculated using the Quadrant Magnetics Calculator^13^, which was developed using Parker’s Equations^14^ for the *P*_*c*_ (= *B*_*d*_*/H*_*d*_), and (*B*_*d*_, *H*_*d*_) is the working point of a stand-alone magnet. The *P*_*c*_ values calculated from Parker’s equations have minor differences compared with those from several other reports^15–18^. The *P*_*c*_ values can derive the demagnetization factor *N*, using the equation shown below (*P*_*c*_ < 0 since *H*_*d*_ is in the 2nd quadrant).

3 $$P_{c} \, = \,B_{d} \,/\,H_{d} \, = \,{1}\, - \,{1}\,/\,N,\,\,\,{\text{i}}{\text{.e}}{.}\,\,\,\left| {P_{c} } \right|\, = \,1\,/\,N\, - \,1$$

Pugh et al.^19^ calculated the *N* for cylinder magnets with various ratios of length/diameter using 3D FEA simulations, and compared all these reported *N* values (i.e., the *P*_*c*_ values), which showed some differences among all the reports. Parker’s equations were used by the Quadrant Magnetics Calculator^13^ and our previous paper^6^ since the equations cover various magnet shapes.

### FEA simulations for interactive force verification and LD level calculation

FEA simulation was carried out using the newest version 2022.1 of Simcenter MagNet, which enabled the permanent magnets to be simulated involving the BH curve in the 3rd quadrant for the first time^20^. This is important for accurately calculating the force in a small distance, as the BH curve of N55 is nonlinear in the 2nd and 3rd quadrants. The interaction of N55 like poles at a small distance involves the demagnetizing field in the 3rd quadrant^8^. MagNet version 2018 is also used for simulating magnetic flux distributions.

FEA simulation is used to obtain the interactive forces between the poles, and to verify the attracting conditions of like magnetic poles. FEA 3D models were set with a mesh of 0.5 mm in the targeted areas. The magnetization directions of the two magnet poles were set opposite to each other.

Besides the interactive forces, the simulations also result in magnetic field distributions across the models, and the magnetic field in any location in the model can then be extracted from the simulated magnetic field. The *LD* level, *LDL*, can be determined in the following four steps:

Step 1 Extracting the magnetic field strength in certain locations from the FEA simulated results;

Step 2 Integrating the area of B_z_*LD*_ covered by the curve of magnetic field with *LD* vs. location;

Step 3 Integrating the area of B_Z_*No-LD*_ covered by the curve of magnetic field without *LD* vs. location;

Step 4 The LD level *LDL* = (B_z_*LD*_—B_z_No-*LD*_)/ B_z_No-*LD*_.

## Data Availability

The datasets generated during and/or analyzed during the current study are available from the corresponding author on request.
